# Regulation of the Ras-Related Signaling Pathway by Small Molecules Containing an Indole Core Scaffold: A Potential Antitumor Therapy

**DOI:** 10.3389/fphar.2020.00280

**Published:** 2020-03-13

**Authors:** Fei-Yu Chen, Xiang Li, Hong-Ping Zhu, Wei Huang

**Affiliations:** ^1^State Key Laboratory of Southwestern Chinese Medicine Resources, School of Pharmacy, Chengdu University of Traditional Chinese Medicine, Chengdu, China; ^2^Antibiotics Research and Re-evaluation Key Laboratory of Sichuan Province, Sichuan Industrial Institute of Antibiotics, Chengdu University, Chengdu, China

**Keywords:** antitumor, indole, Ras-Related signaling pathway, screening and optimizing, regulatory mechanisms

## Abstract

The Ras-Related signaling pathway plays an important role in cell development and differentiation. A growing body of evidence collected in recent years has shown that the aberrant activation of Ras is associated with tumor-related processes. Several studies have indicated that indole and its derivatives can target regulatory factors and interfere with or even block the aberrant Ras-Related pathway to treat or improve malignant tumors. In this review, we summarize the roles of indole and its derivatives in the isoprenylcysteine carboxyl methyltransferase-participant Ras membrane localization signaling pathway and Ras-GTP/Raf/MAPK signaling pathway through their regulatory mechanisms. Moreover, we briefly discuss the current treatment strategies that target these pathways. Our review will help guide the further study of the application of Ras-Related signaling pathway inhibitors.

## Introduction

Cancer poses a great threat to human health, and numerous oncogenes have been identified. Ras, one of the most common proto-oncogenes in human cancer, plays an important role in tumor development. About 30% of human malignancies are associated with Ras mutations (Hunter et al., 2015). The three Ras isoforms, namely, K, H, and N-Ras, belong to the Ras gene family, which is a member of the Ras protein superfamily. Canonically, superfamily Ras proteins exist in two states: the GTP-bound “on” state and GDP-bound “off” state. The transformation of Ras proteins depends on GTPase activating protein (GAP) and guanine nucleotide exchange factors (GEFs). Mutated Ras proteins initiate cell transformation, drive mutant oncogenesis, and promote tumor maintenance. Therefore, inhibitors with high affinity, bioavailability, and low toxicity are urgently needed to control tumor growth and metastasis.

**FIGURE 1:**
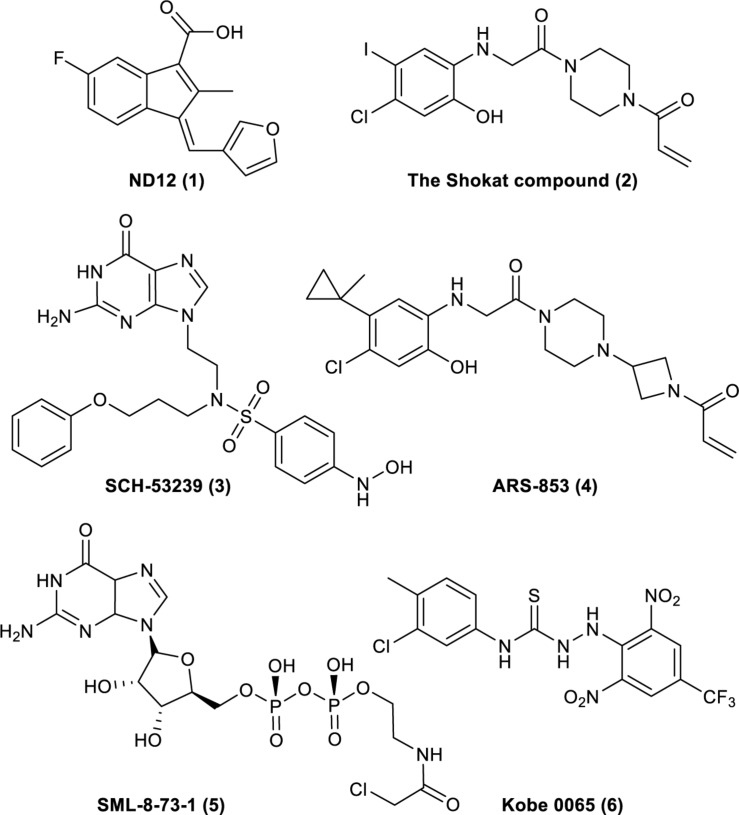
Compounds that have been reported to bind to Ras. (1). ND12 is a sulindac derivative that blocks the growth of Ras-transformed cells (Karaquni et al., 2002a, b). (2). The Shokat compound was identified by using a disulfide fragment-based screening approach with GDP-bound K-Ras (G12C) (Ostrem et al., 2013). (3). SCH-53239 was designed to inhibit guanine nucleotide exchange (Taveras et al., 1997). (4). ARS-853 is a selective covalent inhibitor of K-Ras G12C that inhibits mutant K-Ras–driven signaling by binding to the GDP-bound oncoprotein and preventing activation (Patricelli et al., 2016). (5). SML-8-73-1 can bind to K-Ras (G12C) (Lim et al., 2014). (6). Kobe 0065 was selected for its ability to inhibit the binding of H-Ras–GTP to Ras–Raf-binding domains (Shima et al., 2013).

The understanding of Ras’s biochemical properties and crystal structure continues to deepen with the development of computer modeling technology and drug screening technology. Several new breakthroughs have been made in research on the direct targeting of Ras proteins (Figure 1; Taveras et al., 1997; Karaquni et al., 2002a, b; Ostrem et al., 2013; Shima et al., 2013; Lim et al., 2014; Patricelli et al., 2016). Currently available strategies for deploying the Ras-Related signaling pathway in antitumor drug treatment mainly include directly targeting Ras proteins, inhibiting processes related to translation or modification, and inhibiting the downstream molecules of the Ras-Related signaling pathway (Tamanoi and Lu, 2013; Cox et al., 2014). Thus, the Ras-Related signaling pathway represents a new direction for developing effective and accurate antitumor drugs, and research on antitumor drugs based on Ras is crucial.

**FIGURE 2:**
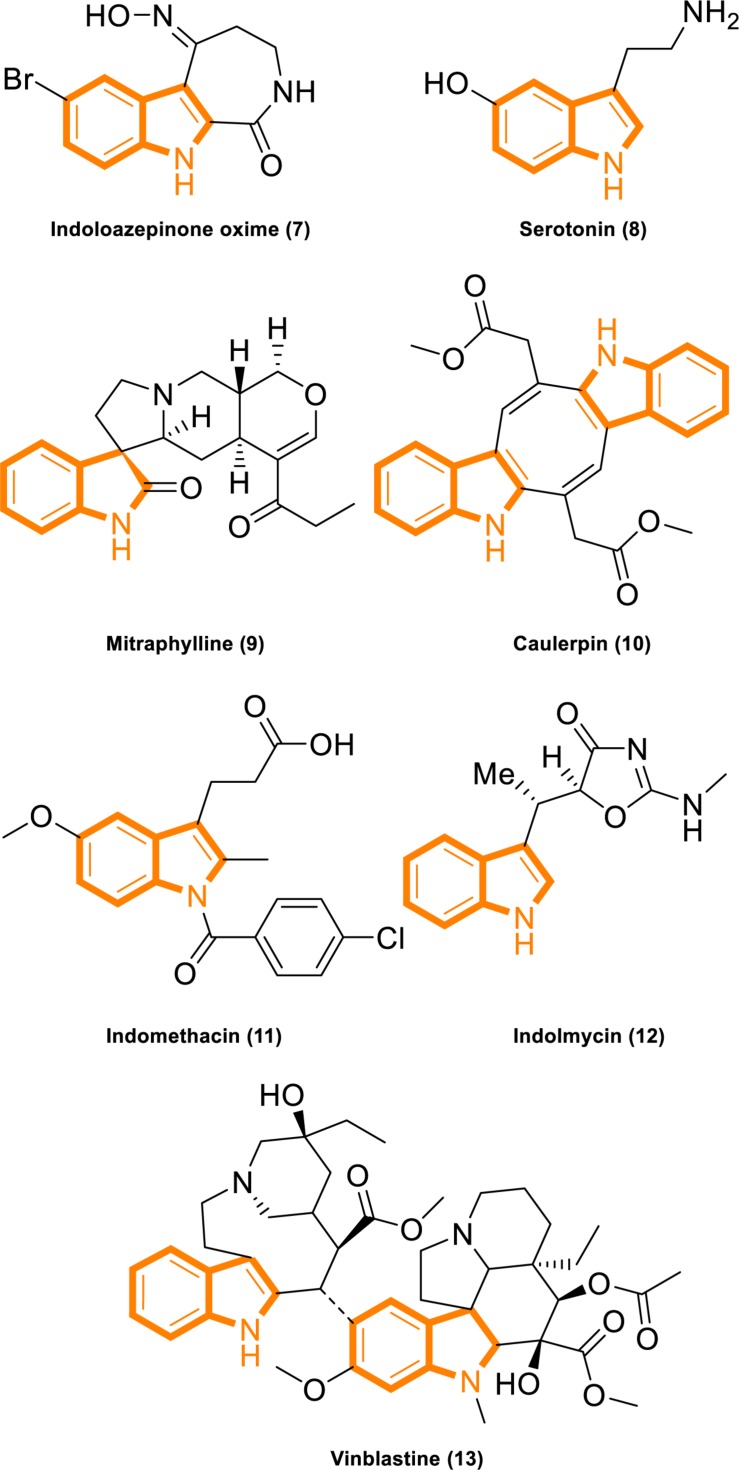
Indole derivatives with characterized activity. (7). Indoloazepinone oxime is derived from marine products with anticancer properties (White et al., 2012). (8). Serotonin is a neurotransmitter with activities that modulate central and peripheral nervous system functions (Watanabe et al., 2011). (9). Mitraphylline activates neutrophils that contribute to the attenuation of inflammatory episodes (Montserrat-de la Paz et al., 2016). (10). Caulerpin synergizes with the glycolytic inhibitor 3BP to inhibit cellular proliferation *in vitro* and *in vivo* (Yu H. et al., 2017). (11). Indomethacin is a non-steroidal anti-inflammatory drug (Nalamachu and Wortmann, 2014). (12). Indolmycin is a potential topical agent for the control of staphylococcal infections (Hurdle et al., 2004). (13). Vinblastine is an antineoplastic agent (Haque et al., 2018).

Indole is widely found in cruciferous plants and bacteria, and several indole derivatives are closely related to life activities. It plays important roles as an interspecies and interkingdom signaling pathway molecule in a wide range of antiinflammatory and antitumor activities, with minimal side effects and strong selectivity (Järvisalo and Saris, 1975; Banerjee et al., 2011; Chripkova et al., 2016; Huang et al., 2016; Patil et al., 2016; Okada et al., 2017; Singh et al., 2017; Yang et al., 2017; Yu C. P. et al., 2017; Hashmi et al., 2018; Zhao et al., 2018). Popular active natural and synthetic indole derivatives have been discovered and investigated (Figure 2; Hurdle et al., 2004; Watanabe et al., 2011; White et al., 2012; Nalamachu and Wortmann, 2014; Montserrat-de la Paz et al., 2016; Yu H. et al., 2017; Haque et al., 2018). Meanwhile, the molecular design and synthesis of indoles have attracted extensive attention, and the structural modification of indole have become an important direction for screening compounds with novel structures and strong antitumor activity. Therefore, using indoles as lead compounds in the synthesis and screening of antitumor drugs with a broad range of targets and high selectivity has wide research prospects.

The occurrence of tumors is related to the abnormal activation of intracellular signaling pathways and involves the malignant proliferation, invasion, and metastasis of cells. A series of studies demonstrated that indole derivatives can target Ras proteins or Ras-related proteins through multiple pathways and block the transmission of the Ras-Related signaling pathway. The relationship between indole derivatives and the Ras-Related signaling pathway was first reported in 2005 (Winter-Vann et al., 2005). Since then, numerous small molecules based on indoles that inhibit the Ras-Related signaling pathway by targeting various molecular targets within the Ras-Related signaling pathway have been reported. In this review, we provide a summary of the application of indole derivatives for inhibiting the Ras-Related signaling pathway and our opinions on this approach. We hope this work will provide useful clues for the rational design of indole-containing compounds as highly potent Ras-Related signaling pathway Inhibitors and provide a reference for the development and research of antitumor drugs.

## Indole Derivatives as Ras-Related Signaling Pathway Inhibitors

**FIGURE 3:**
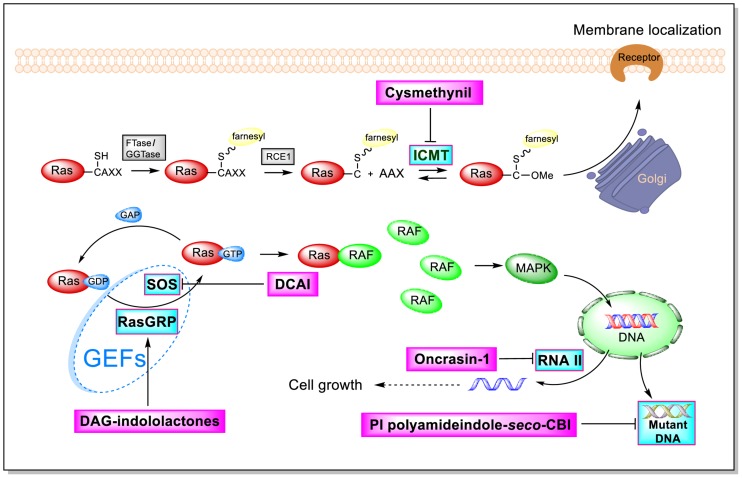
Influence of indole derivatives on the Ras-Related signaling pathway.

As shown by the illustration of the Ras-Related signaling pathway in Figure 3, indole-based inhibitors of the Ras-Related signaling pathway affect five different molecular targets. Indole derivatives affect the Ras-Related signaling pathway by (1) inhibiting Icmt action; (2) inhibiting SOS-mediated exchange action; (3) affecting RasGRP action; (4) inhibiting the phosphorylation of the C-terminal domain (CTD) of RNA polymerase II; and (5) inhibiting mutant DNA action.

### Isoprenylcysteine Carboxyl Methyltransferase Inhibition

Isoprenylcysteine carboxyl methyltransferase (Icmt) can methylate the carboxyl-terminal isoprenylcysteine of CAAX proteins, such as Ras and Rho proteins. The targeting of Ras proteins to the plasma membrane is closely related with its carboxyl methylation. Thus, Icmt may greatly affect the delivery of Ras proteins. Young’s group showed that the inactivation of Icmt blocks transformation by an oncogenic form of B-Raf (V599E) even though the effect of inactivating Icmt is not limited to the inhibition of K-Ras-induced transformation (Bergo et al., 2000, 2004). A previous work on Icmt genetic disruption demonstrated that Ras proteins are mislocated and tumorigenesis is drastically reduced in cells lacking Icmt. And Icmt is not only reducing the growth of K-Ras- but also can reduce the growth of B-Raf-. Therefore, Icmt is a potential target for inducing malignancies. These studies provided strong evidence that blocking Icmt activity has profound consequences for oncogenic transformation (Bergo et al., 2000; Baron and Casey, 2004).

**FIGURE 4:**
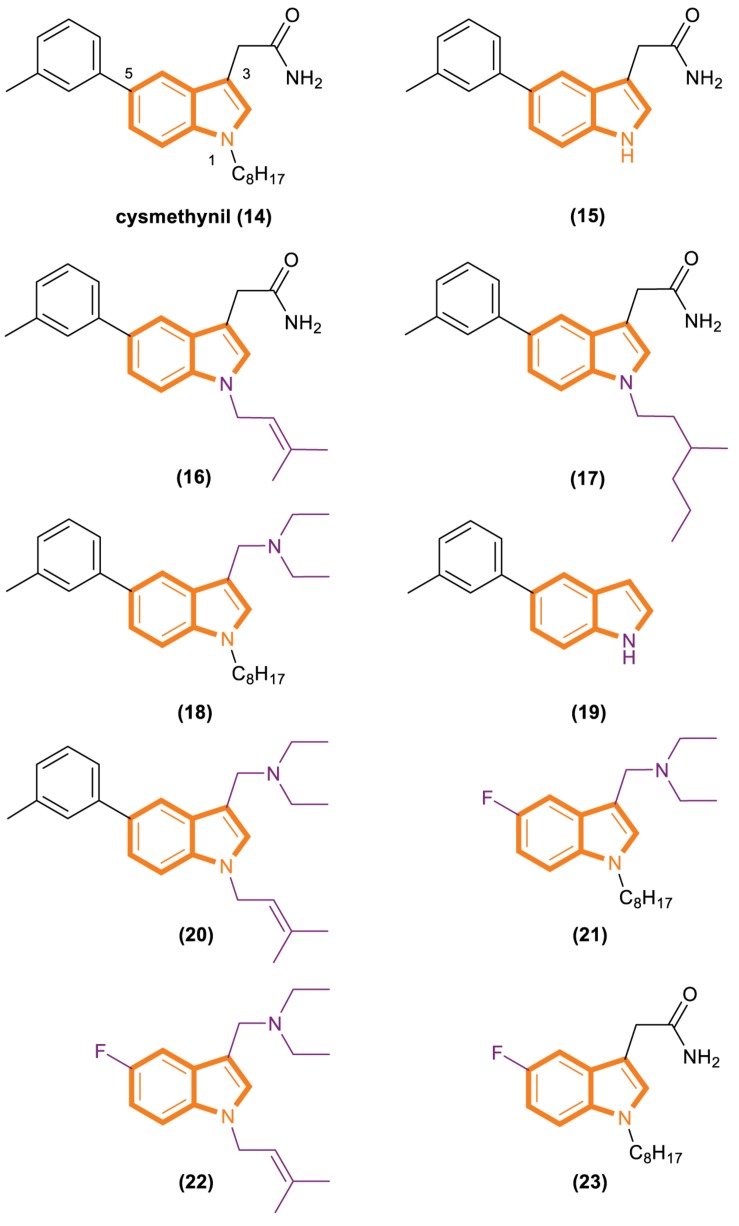
Cysmethynil and its analogs (14–23).

In 2005, Casey’s group reported that cysmethynil (Figure 4), a small-molecule indole Icmt inhibitor, can be used to treat cancer cells (Winter-Vann et al., 2005). Cysmethynil treatment can lead the inhibition of cell growth in an Icmt-dependent way. This cell growth phenomenon Indicates the mechanism-based activity of this indole derivative. In particular, treating cancer cells with cysmethynil results in the mislocalization of Ras and it impairs the epidermal growth factor signaling pathway. In a human colon cancer cell line, cysmethynil treatment blocks anchorage-independent growth. On the contrary, Icmt overexpression reversed the influence. These findings, together with the discovery of enzymatic gene disruption, indicate that Icmt inhibitors may have strong therapeutic potential.

A series of kinetic experiments has been designed to classify the substrate-related inhibition patterns of cysmethynil. The data obtained from Casey’s experiment show that cysmethynil is a competitive inhibitor relative to the farnesylated protein substrate (Baron et al., 2007). In the assay conditions, they calculated Ki was 2.2 ± 0.5 μM. Km determined for K-Ras was 2.9 μM, and the value of Vmax was 65.8 pmol/h. When FK-RasCOO^–^ and cysmethynil concentrations stay fixed, the AdoMet concentration was varied. Meanwhile, the characterization of the inhibition of FK-RasCOO^–^ methylation by using Lineweaver–Burk plot got the lines which intercept at a negative value on the *x*-axis. It preliminarily indicateed that inhibition by the compound was not competitive with respect to AdoMet. The Ki value is 1.9 ± 0.2 μM and Km for AdoMet is 7.1 μM. And given that the kinetic mechanism for Icmt proceeds with AdoMet binding first (Shi and Rando, 1992; Baron and Casey, 2004), this inhibition pattern indicated that either the free enzyme or the AdoMet–Icmt complex was strongly associates with the inhibitor.

Additional experiments have revealed that cysmethynil is a competitive inhibitor relative to the isoprenyl cysteine substrate and is a non-competitive inhibitor relative to the methyl donor AdoMet in the reaction (Baron et al., 2007). Therefore, it is a classic time-varying inhibitor of Icmt. Finally, they analyzed the time-dependent condition of the cysmethynil-induced inhibition of Icmt, and the reversibility of the time dependence of cysmethynil on enzymatic activity was evaluated. Cysmethynil can be classified as a slow-binding inhibitor of Icmt, and further studies confirmed that the inhibition of cysmethynil is reversible.

Cysmethynil was previously identified as the most potent compound in a library of cysmethynil compounds, so it was selected for further study. A previous work examined a select set of these compounds to determine whether they exhibit time-dependent inhibition and found that the substituent on the indole nitrogen plays an important role in this behavior (Baron et al., 2007). The inhibition potential of indoles containing various R1 substituents was detected under standard conditions or after Icmt incubation at 37°C for 30 min. A close correlation among the lipophilicity induced by R1 (Figure 4), positional changes, and IC50 values was observed. Specifically, when the R1 substituent is an isobutyl or cyclopropyl, the IC50 value of the compound is the same whether or not it is preincubated with enzymes. However, time-dependent inhibition was observed when the R1 substituent was changed to a longer chain or highly hydrophobic group [such as hexyl, octyl (present in cysmethynil), benzyl, 3-trifluoromethyl benzyl, or naphthyl]. Moreover, the time dependence of indole-based Icmt inhibitors decreases as the length of indole nitrogen substituents shortens. Thus, the substituents on the indoles of these Icmt inhibitors are the key to time-dependent inhibition.

Another experiment was performed to systematically study the influence of structural modification on the activity of cysmethynil. Several cysmethynil derivatives were identified, and their SlogP values, which test the logarithm of the octanol/water partition coefficient of the compound in the protic state; Icmt inhibitory activities; and antiproliferative activity against breast cancer MDA-MB-231 cells were evaluated (Winter-Vann et al., 2005; Go et al., 2010). The main contribution of the position of R1 is related to the length and hydrophilic or lipophilicity of the attached side chain. When the R1 position is not replaced (Figure 4, 15), the SlogP value is 3.2, the IC50-Icmt inhibition value is 33 ± 10 μM (mean and SD of three or more determinations), and the IC50-antiproliferative activity is >100 μM. When the R1 position is replaced with octyl (Figure 4, 16), the SlogP value is 6.3, the IC50-Icmt inhibition is 1.5 ± 0.2 μM, and the IC50-antiproliferative activity is 21.8 ± 0.8 μM. Geranyl (Figure 4, 19), a feasible alternative to octyl (present in cysmethynil), has a SlogP value of 6.6, IC50-Icmt inhibition value of 1.1 ± 0.2 μM, and IC50-antiproliferative activity of 10.6 ± 0.7 μM. Isoprenyl (Figure 4, 17) retains good activity (including antiproliferative activity). Although its IC50-Icmt inhibition value is 7.7 ± μM and its IC50-antiproliferative activity is 28.5 ± 5.3 μM, its SlogP value is 4.9. These results indicate that if the R3 position have an appropriate amino groups, the chains with low lipophilicity will improved activities in Icmt inhibition and cell growth inhibition (Lau et al., 2014).

There is an important finding in the experiment that the critical role of the R3 position may influence Icmt inhibition and cell viability. However, introducing an amino functionality improves activities under most conditions. The data show that compound 18 (Figure 4) has a SlogP value of 6.9, IC50-Icmt inhibition value of 0.7 ± 0.1 μM, and IC50-antiproliferative activity of 3.6 ± 0.1 μM. Compound 20 (Figure 4) has a SlogP value of 5.5, IC50-Icmt inhibition value of 2.4 ± 0.2 μM, and IC50-antiproliferative activity of 3.8 ± 0.3 μM. Thus, compound 26 (Figure 4) is superior among the tested compounds.

Substituents at the R5 position have the least effect on Icmt inhibition and exhibit incremental changes during modification. Such alterations may be beneficial because they can modulate the function of physicochemical properties while having minimal adverse effects on activity. Compound 21 (Figure 4) has a SlogP value of 5.1, IC50-Icmt inhibition value of 4.1 ± 1.1 μM, and IC50-antiproliferative activity of 7.2 ± 0.4 μM. Introducing a fluoro atom at the R5 position causes a desired reduction in lipophilicity without a drastic loss in Icmt inhibitory activity. By contrast, the effects of compounds 22 and 23 (Figure 4) are unsatisfactory. Compound 22 has a SlogP value of 3.7, IC50-Icmt inhibition value of 35 ± 6 μM, and IC50-antiproliferative activity of 32 ± 2 μM. Compound 23 has a SlogP value of 4.4, IC50-Icmt inhibition value of 7.0 ± 3.4 μM, and IC50-antiproliferative activity of 70 ± 20 μM.

**FIGURE 5:**
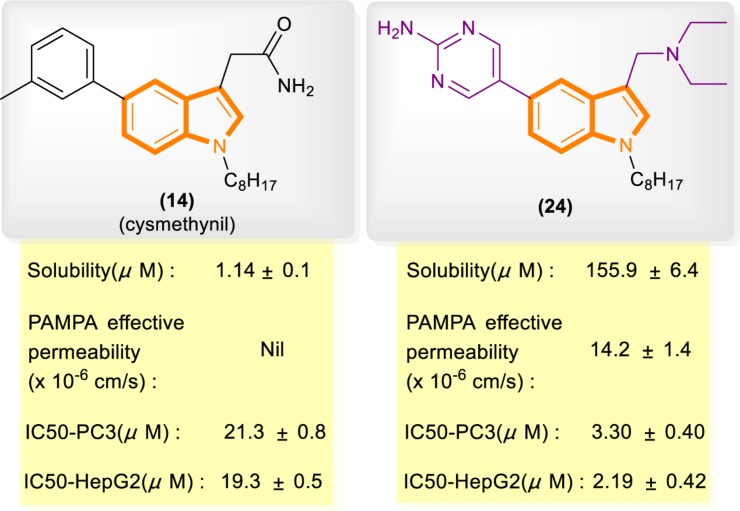
Comparison of some aspects of cysmethynil and 24.

In reference to a similar study on the cysmethynil indole nucleus, Wang and Go’s group modified cysmethynil to improve its drug-likeness while retaining its activity against Icmt (Ramanujulu et al., 2013; Lau et al., 2014). They reported compound 24 (Figure 5), which has excellent physical properties and can considerably improve the curative effect. Compound 24 is highly water soluble and has good cell permeability. The IC50 values of 24 for the inhibition of HepG2 and PC3 cell growth are almost 10-fold lower than those of cysmethynil.

Though we’re gradually finding out the influence of Icmt deficiency on cell growth and tumorous transformation, Icmt participating in protein modifications still remains a vast platform to explore (Bergo et al., 2004). Meanwhile, some interesting reports showed there is a possibility that sometimes due to lacking of Icmt, a Ras-independent pathway functions to catalyze growth factor–induced activation of ERK-related signal pathway (Clarke and Tamanoi, 2004).

Overall, these meaningful findings can assess the potential of targeting this enzyme in anticancer drug design, and markedly enhance our understanding about the mechanism of Icmt inhibition by compounds containing indole core scaffold. At the same time, specific Icmt inhibitors have been developed as pharmacological tools and potential drugs for the treatment of cancer. Screening studies have shown that the superior compound 21 is equivalent to cysmethynil in terms of Icmt inhibition and has strong antiproliferative activity against MDA-MB-231 cells. Therefore, the results of these studies can be used to guide future synthetic work with the aim of obtaining clinically useful effective inhibitors of the Icmt steroid.

### Inhibition of SOS-Mediated Activation

**FIGURE 6:**
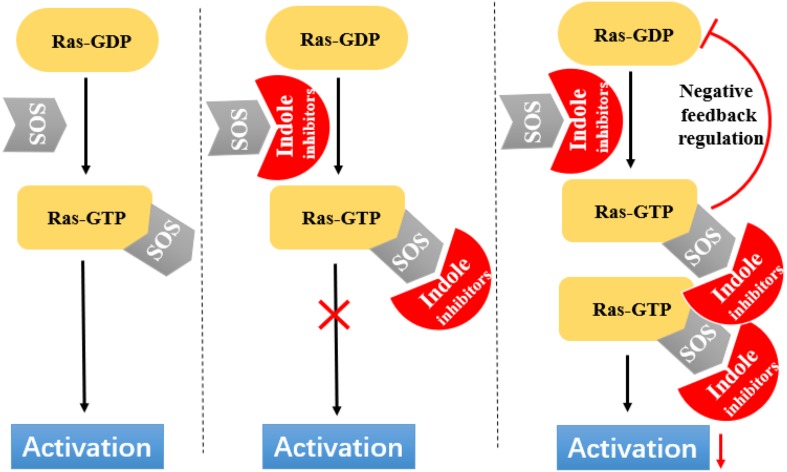
Indole derivatives interfere with Ras activation by inhibiting SOS-mediated nucleotide exchange activity.

Ras circulates between inactive (GDP bound) and active (GTP bound) states and delivers cellular signals in response to extracellular stimuli. Ras activation is strictly catalyzed by GEFs. Under the action of extracellular signals, Ras is catalyzed by GEFs, especially the SOS1 protein. During nucleotide exchange, Ras interacts with SOS1 protein to form a complex: Ras–SOS1–Ras. It changes from the GDP-bound (Ras-GDP) state to GTP-bound (Ras–GTP) state. Upon activation, Ras interacts with effector proteins, such as Raf kinase, to promote cell growth and survival. Subsequently, intrinsic Ras enzymes hydrolyze GTP into GDP to terminate the Ras-Related signaling pathway (Figure 6).

**FIGURE 7:**
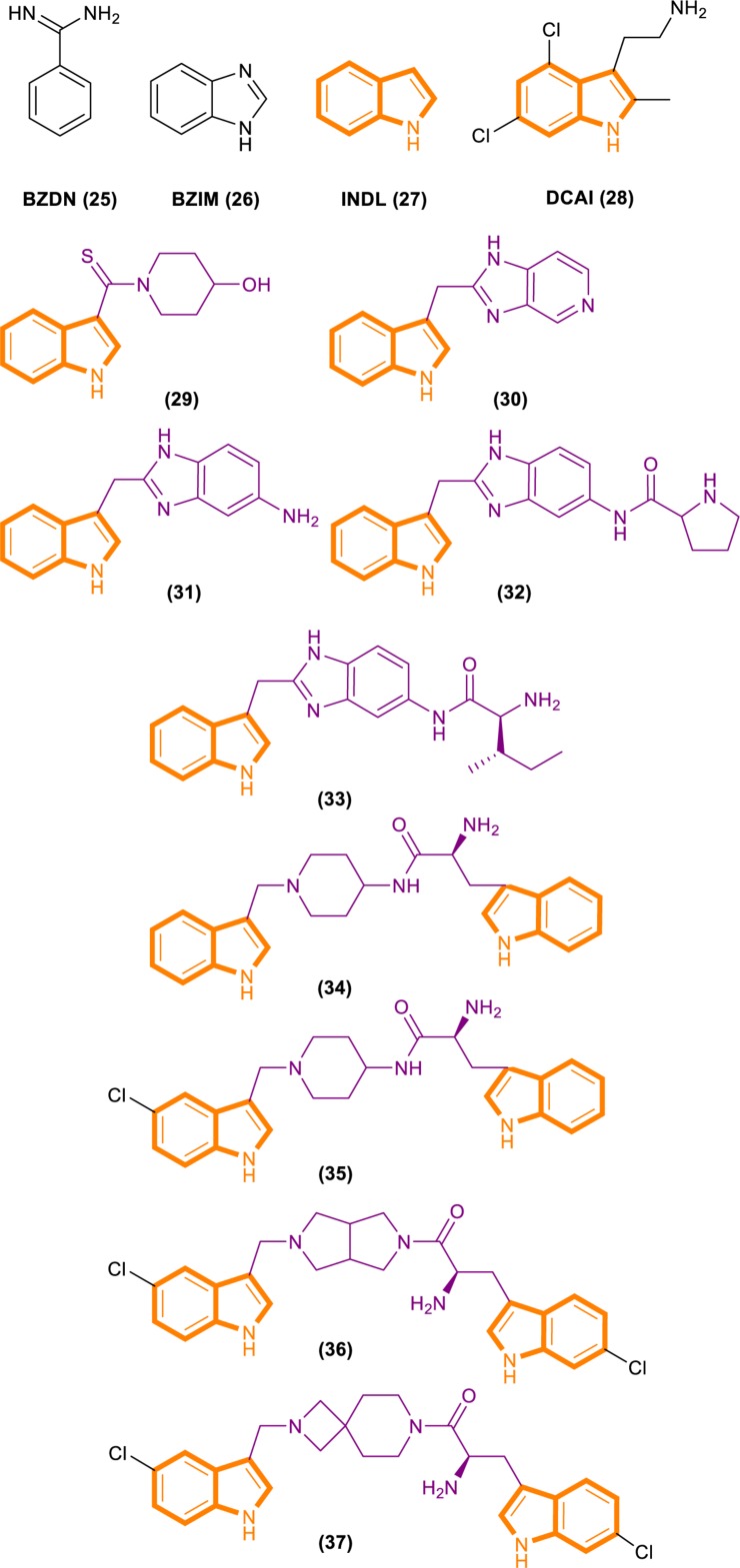
Structures of BZDN, BZIM, INDL, DCAI, Benzimidazole-indole analogs (29–33) and Aminopiperidine–indole analogs (34–37).

Fang’s group identified a group of small molecules that bind to a common site on Ras by using NMR-based fragment screening (Freedman et al., 2006; Maurer et al., 2012). Structural analysis predicted that compound binding can interfere with Ras–SOS1 interactions. A titration experiment showed that DCAI (Figure 7) blocks nucleotide exchange and release nucleotide exchange, with the IC50 values of 342 ± 22 μM and 155 ± 36 μM. BZIM (Figure 7) has little or no effect on nucleotide exchange and release. Further structural analysis of the apoRas–SOS complex has shown that the binding of DCAI and K-Ras inhibits SOS-mediated catalysis and interferes the formation of intermediates during its reaction. Moreover, the sequence and structural homology between SOS1 and Ras-GEF1, indicate that DCAI might also inhibit other Ras–GEFs.

Through fragment-based screening, Fesik and his co-workers found small molecules that bind directly to K-Ras, inhibit the activation of SOS-catalyzed K-Ras, and optimize their synthesis analogs (Sun et al., 2012). Tests for the indole-benzimidazoles’ KD value (KD values were measured from the changes in chemical shifts observed in the HSQC spectra of uniformly 15N-labeled protein as a function of added compound) and SOS catalytic inhibition rate (the inhibition percentage of SOS-catalyzed nucleotide exchange observed using 1 mm compound) revealed that compound 33 (Figure 7) had good effect. The compound has a KD value of 190 μM, and the inhibition rate of SOS catalytic nucleotide exchange is 78% ± 8%. Compound 31 (Figure 7) has KD > 1300 μM and does not inhibit SOS catalytic nucleotide exchange. Compound 32 (Figure 7) has a KD value of 340 μm and inhibition rate of SOS catalytic nucleotide exchange of 58% ± 8%.

Through fragment-based screening, Fesik’s laboratory identified compounds derived from an aminopiperidine indole scaffold that can create a negative feedback mechanism after binding to the CDC25 domain of SOS1 (Burns et al., 2014; Howes et al., 2018). The reaction of the compound is mainly divided into the early reaction and late reaction stages: They initially bind with SOS1, thereby stimulating the exchange of GTP and GDP on Ras. This stage is characterized by the rapid induction of Ras–GTP levels. Early increases in Ras–GTP enhance the downstream signaling pathway as demonstrated by the increased phosphorylation of ERK. Subsequently, a negative feedback loop leads to the phosphorylation of SOS1 by the active form of ERK at the c terminal. This phenomenon results in the decentralization of SOS1 from near Ras and the gradual reduction in Ras–GTP over time result in the decline of the phosphorylation of ERK in the late stage of the compound reaction.

The ability of these compounds to affect SOS1-mediated nucleotide exchange was assessed by using a high-throughput nucleotide exchange assay (Burns et al., 2014, 2018; Abbott et al., 2018). Data analysis showed that compound 34 (Figure 7) had an EM50 (*in vitro* compound potency was defined as the half maximal effective concentration (Abbott et al., 2018).) value of 8.97 ± 12.34 μM and Max.Act. (compound efficacy *in vitro* was expressed in terms of maximal percent activation, and activation values represent percentage relative to DMSO control at a ligand concentration of 100 μM (Abbott et al., 2018).) value of 641% ± 255.3%. Compound 35 (Figure 7) has an EM50 value of 9.6 ± 4.27 μM and Max.Act. value of >1000%. Compounds 36 (Figure 7) and 37 (Figure 7) demonstrated good effects after optimization. Compound 35 had an EM50 value of 0.8 ± 0.47 μM and Rel.Act. (Rel.Act. values calculated as the percentage activation for each compound at 100 μM relative to the activation of control compound 35 at 100 μM (Abbott et al., 2018).) value of 119% ± 16.3%. Compound 36 had an EM50 value of 0.8 ± 0.36 μM and Rel.Act. of 78% ± 23.3%. So compound 36 and 37 had a positive affect with SOS1-mediated nucleotide exchange.

The recognition and characterization of the binding sites of the Ras–SOS–Ras complex represent another innovative method for research on targeting Ras signals. SOS, a key control point in the activation of various Ras subtypes and RTKRas signal transduction, is a promising intervention point for the treatment of Ras-driven cancer (Burns et al., 2014). Further medicinal chemistry will take efforts to control selectivity and enhance potency. Given that small G proteins share similar structural features, we are supposed to sufficiently understand the potential pathways that these small molecule inhibitors target and the regulatory mechanisms of these biologically important regulatory proteins, to help discover K-Ras inhibitors for cancer.

### Selective Activators of RasGRP

**FIGURE 8:**
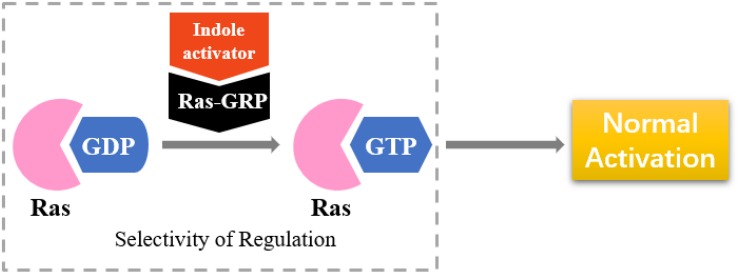
Selectivity of regulation by indole derivatives.

RasGRP is an activator of small GTPases in the Ras family (GEFs), and it is prominently expressed in blood cells (Figure 8). RasGRP malfunction may lead to autoimmunity and contribute to hematologic malignancies (Stone, 2011). RasGRP1 has been demonstrated to play a key role in skin cancer (Sharma et al., 2014). Other studies have shown that RasGRP is a target of the anticancer drug ingenol-3-angelate (Song et al., 2013).

**FIGURE 9:**
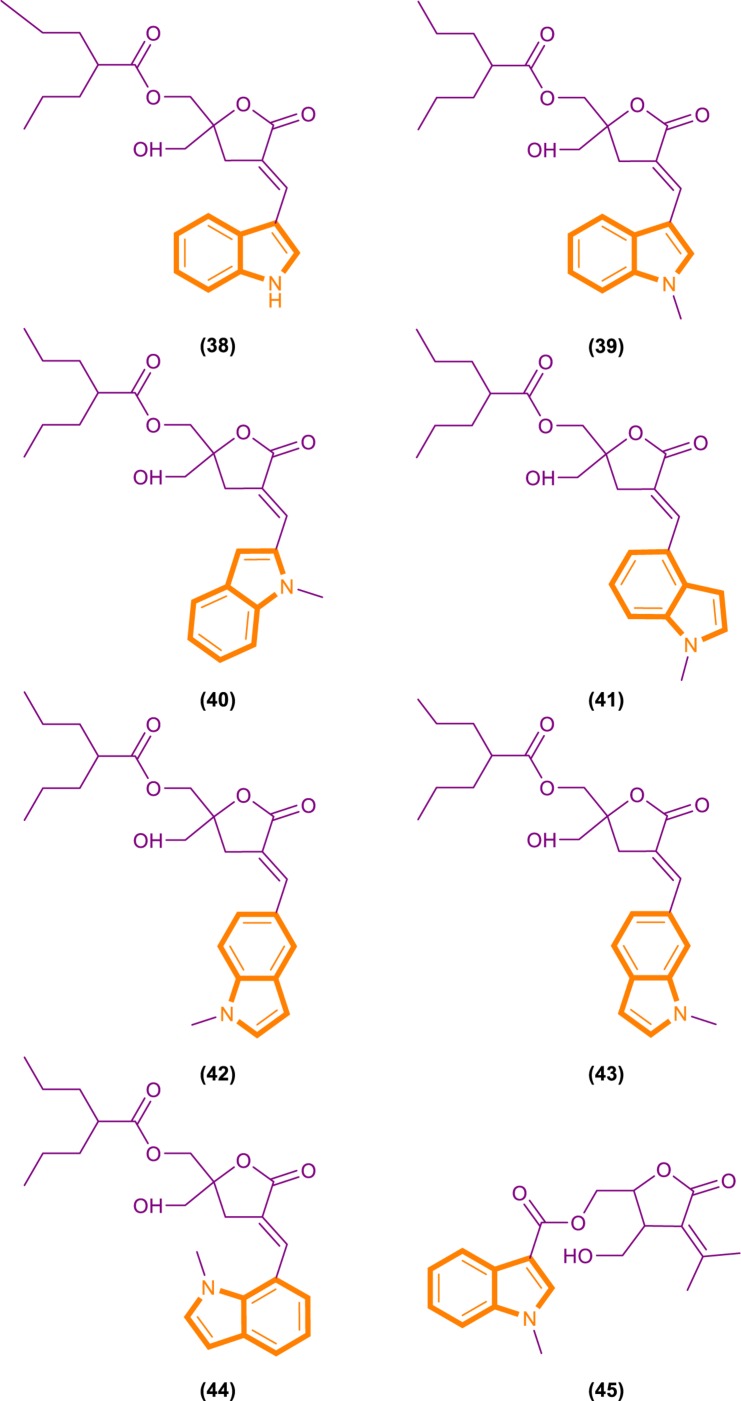
DAG-indololactone analog (38–45).

On the basis of the interaction between cell analysis and biophysical analysis with a model membrane, Jelinek and Comin’s groups synthesized DAG-indololactone analogs, wherein indole rings are linked to DAG in different positions (Garcia et al., 2014; Elhalem et al., 2017). Compound 39 (Figure 9), which has an indole substituted at the R3 position, presents excellent binding affinity and selectivity to RasGRP1 as evidenced by its ClogP value of 5.0 and selectivity for PKCα, PKCε, and RasGRP1 [the binding affinities of compounds for PKCα, PKCε, and RasGRP1 were determined *in vitro* by competition with bound (20-3H)phorbol 12,13-dibutyrate (PDBU) in the presence of 100 μg/mL phosphatidylserine (Elhalem et al., 2017)]. Its PKCα/RasGRP1 is 22, and its PKCε/RasGRP1 is 53. By contrast, compound 45 (Figure 9) has a ClogP value of 2.6 and PKCα/RasGRP1 and PKCε/RasGRP1 values of only 1.08 and 0.4, respectively.

RasGRP family members have the important biological effects, and selective reagents can interact with the specific C1 domain of RasGRP. Experimental data have shown that compound 39 is useful for activating proteins that contain a C1 domain and is the most selective RasGRP activator. Therefore, the synthesis of these compounds (such as compound 39) can provide evidence for the research of related drugs that interfere with and control the Ras-Related signaling pathway.

### Inhibition of the Phosphorylation of the CTD of RNA Polymerase II

Tumor-selective anticancer drugs induce cell death selectively based on synthetic lethality (Torrance et al., 2001; Dolma et al., 2003; Guo et al., 2008). Various investigators have applied synthetic lethality screening to identify genes that are crucial for the survival of certain oncogene-transformed cells or those that sensitize cells to chemotherapy (Whitehurst et al., 2007; Luo et al., 2009; Scholl et al., 2009) or small molecules that selectively induce cell death in a subset of oncogene-transformed cells (Torrance et al., 2001; Guo et al., 2008).

**FIGURE 10:**
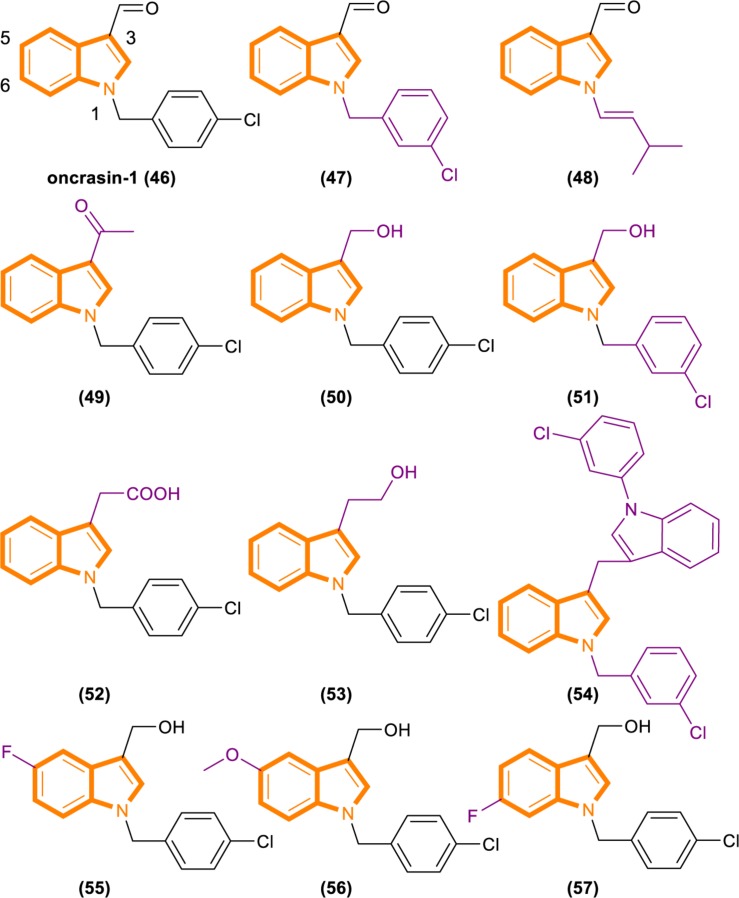
Oncrasin-1 and its analogs (46–57).

Fang’s group recently identified a novel anticancer agent, oncrasin-1 (Figure 10), through synthetic lethality screening on isogenic human ovarian epithelial cells with or without oncogenic Ras expression (Guo et al., 2008; Wu et al., 2011). Oncrasin-1 suppressed the phosphorylation of the largest subunit of RNA polymerase II, COOH-terminal domain (CTD) (Guo et al., 2009). CTD phosphorylation is essential for efficient transcription elongation and recruitment of mRNA processing factors (Archambault et al., 1997; Kim et al., 1997; McCracken et al., 1997; Misteli and Spector, 1999; Bird et al., 2004; Millhouse and Manley, 2005). Inhibiting the function of CTD phosphorylation will disrupt RNA polymerase II function and promotes cell death at last (Chen et al., 2005).

By using synthetic lethality screening on isogenic T29 immortalized normal human ovary epithelial cells and the T29 tumorigenic subclone derived (T29Kt1), Oncrasin-1 was identified firstly (Liu et al., 2004). Subsequent testing showed that several lung cancer cell lines, including H460, with K-Ras mutations are highly sensitive to oncrasin-1. Therefore, it is rational for using T29, T29Kt1, and H460 cell lines to evaluate the cytotoxicity and cancer cell selectivity of oncrasin-1 and its analogs (Wu et al., 2011).

Analysis based on the IC50 [IC50 values of oncrasin-1 and its analogs in T29, T29Kt1, and H460 cells. All IC50 values of ≥31.6 μM were recorded as 31.6 μM (Wu et al., 2011)] values showed that variations in the substitutions of the benzyl group did not have a dramatic effect on the cytotoxicity and selectivity of oncrasin-1 analogs. And oncrasin-1 had IC50-T29 value of >31.6 μM, IC50-T29Kt1 value of 2.51 μM and IC50-H460 value of 0.25 μM. Switching to isopropyl results in complete or partial loss of activity of H460 and T29Kt1 cells. Compound 48 (Figure 10) had IC50-T29 value of >31.6 μM, IC50-T29Kt1 value of >31.6 μM and IC50-H460 value of 1.99 μM. With the process of screening, most of the active compounds contain either an aldehyde group or a hydroxymethyl group at the R3 position of the indole. Compounds containing hydroxymethyl group are more active than aldehyde group. Compound 50 (Figure 10) had IC50-T29 value of >31.6 μM, IC50-T29Kt1 value of 0.079 μM, and IC50-H460 value of 0.016 μM. In H460 cells, most hydroxymethyl derivatives are six times more effective than the corresponding aldehydes. In T29K cells, most hydroxymethyl derivatives are 1100 times more effective than the corresponding aldehydes. The R3 position instead of ketone results in a drastic reduction in activity. Compound 49 (Figure 10) had IC50-T29 value of >31.6 μM, IC50-T29Kt1 value of >31.6 μM and IC50-H460 value of >31.6 μM.

The effects of substituents in other positions of the indole vary in accordance with the position and type of the substituent. Indole substitution at the 5-position or 6-position is well tolerated, whereas 5-F substitution produces the best potency. Compound 55 (Figure 10) had IC50-T29 value of 8.12 μM, IC50-T29Kt1 value of 0.16 μM, and IC50-H460 value of 0.07 μM.

Identifying through analog analysis for compound 46 (oncrasin-1, Figure 10), another potent analog compound 51 (Figure 10) was found (Guo et al., 2011). Its anticancer spectrum and activity in the NCI-60 cell line panel are similar to those of compound 46. And *in vivo* activity and safety profiles of compound 51 are superior to those of compound 46. What’s more, compound 51 modulates the functions of multiple pathways, such as RNA polymerase, MAP kinase JNK and JAK/STAT3 pathway. All of these activities have critical roles in tumorigenesis or anticancer therapy.

Limited research has been conducted on small molecular RNA polymerase II inhibitors. However, certain antitumor agents of flavopiridol or seliciclib can elicit antitumor activity by blocking CTD phosphorylation in a few of recent studies (Chao and Price, 2001; MacCallum et al., 2005; Baumli et al., 2008). Thereby, it revealed that inhibiting the CTD function of RNA polymerase II could be a novel antitumor approach. The above screening results can be demonstrated that compound 46, 50, 51, and 55 can inhibit CTD phosphorylation and generate antitumor effect, while no exerting considerable toxic effects on normal cells. It may partly pioneer new antitumor therapies.

### Inhibition of K-Ras Codon 12 Mutants

**FIGURE 11:**
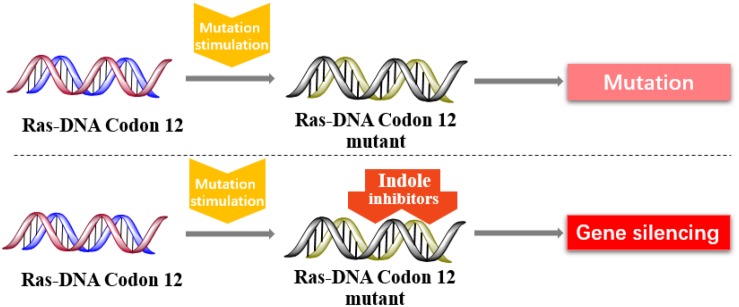
Indole derivatives inhibit the K-RAS codon 12 mutants to silence mutant genes.

**FIGURE 12:**
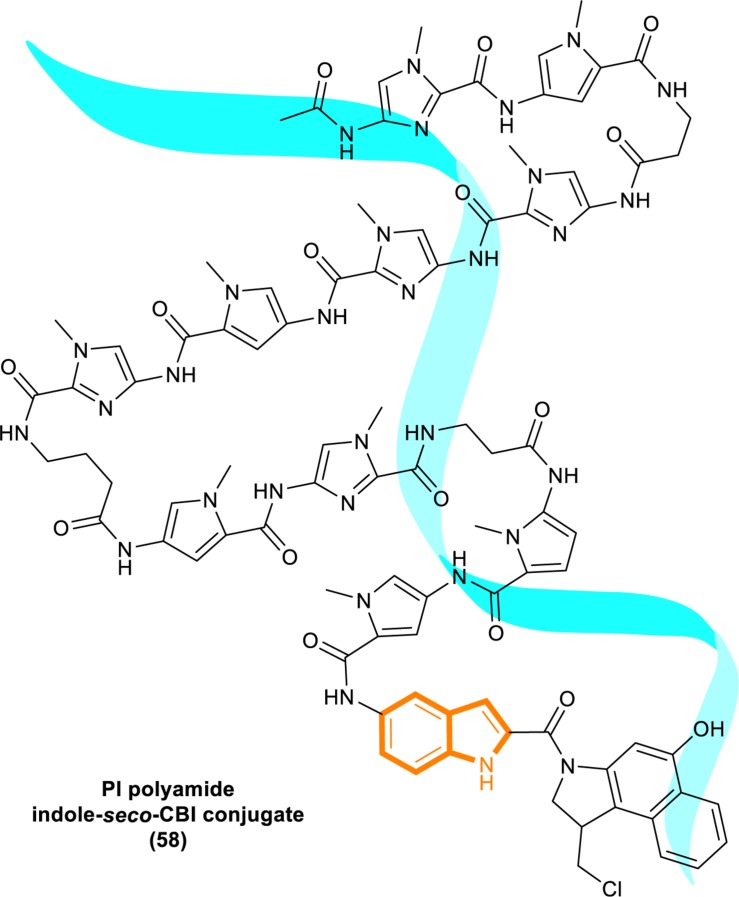
Structure of PI polyamide indole-*seco*-CBI conjugate.

In 2013, Kataoka’s group reported that a small molecule successfully targeted to K-Ras (G12C) mutation *in vitro* (Shima et al., 2013) (Figure 11). Faced with the challenge of developing small-molecule drugs that directly target the mutated K-Ras protein, Nagase’s group synthesized pyrido-imidazole polyamide indole-*seco*-CBI conjugates (PI polyamide indole-*seco*-CBI conjugates) (Figure 12) that target common K-Ras codon 12 mutants (Hiraoka et al., 2015), and they provided a novel approach that directly targets mutant DNA.

Cyclopropylpyrroloindole DNA alkylating agents represent a class of antitumor drugs (Reynolds et al., 1986). But this kind of reagents demonstrates limited DNA sequence selectivity when reacting efficiently. And polyamides are synthetic molecules which can recognize specific sequences in the minor groove of DNA double helix. The DNA affinity of polyamides is similar to DNA-binding proteins (Gottesfeld et al., 2000; Dervan, 2001). Therefore, it is a feasible strategy to design sequence-specific alkylation polyamide conjugates to target sequences to identify specific DNA sequences with high affinity (Wang et al., 2002; Bando and Suqiyama, 2006; Taylor et al., 2014).

The PI polyamine alkylation strategy specifically down-regulates the component activity of K-Ras by inducing DNA damage and apoptosis after cell aging, thereby effectively inhibiting the *in vitro* growth of several K-Ras mutant strains. In the weekly injection of PI polyamide indole-*seco*-CBI conjugates in tumor-bearing mice induced by LS180 or SW480 cells, the *in vivo* growth of K-Ras (G12V/G12D) mutant tumor xenograft was also effectively inhibited (Hiraoka et al., 2015). Therefore, PI polyamide indole-*seco*-CBI conjugates are potential anticancer drug. And applying the technology of PI polyamide-mediated gene silencing may promote the development of molecular targeted therapy strategies.

## Summary and Outlook

In summary, significant progress has been achieved in the discovery of Ras-Related signaling pathway inhibitors containing indole skeletons as new potential antitumor drugs. Indoles, whether derived from natural indole alkaloids or screened and synthesized as highly complex indole derivatives and conjugates, play an important role in the Ras-Related signaling pathway because of their excellent selectivity and stability *in vivo* and *in vitro*. Given that Icmt inhibition in the membrane localization of specific intracellular Ras proteins or the interference of the Ras-GTP/Raf/MAPK pathway or other related pathways processes have been reported, the development of these novel, effective, and safe inhibitors will spur increased interest in the future. The comprehensive comparison of the inhibition mechanisms summarized in this review indicates that SOS-mediated nucleotide exchange may be the best inhibition mechanism. Indole derivatives can participate in this mechanism in two ways to inhibit Ras action: by directly interfering with SOS proteins and through the negative feedback regulation of SOS proteins. The two modes of action show differences but exert good final effects as indicated by experimental results. Therefore, the combination of two indole derivatives can play a synergistic effect on Ras and inhibit the occurrence of Ras-based malignant tumors effectively.

In addition, the incorrect (abnormal) expression of RasGRP, an activator of the GEF family, can lead to the development of malignant tumors. To reduce the occurrence of this vicious cycle, the RasGRP binding affinity and selectivity of selective indole RasGRP activators have been studied. These compounds can restore normal Ras function to a certain extent and reduce misexpression to indirectly inhibit Ras misexpression. Although these studies did not completely achieve direct Ras inhibition, they selected a substance (DAG-indololactones) that can specifically bind to RasGRP activators, and the structure of DAG-indololactones is constantly optimized and modified. Future research should focus on screening compounds that regulate (or inhibit) RasGRP on these bases and play a direct role in the Ras-Related signaling pathway.

Finally, DNA mutations are common in different organisms, and mutations are highly uncertain. A new method with indole conjugates to directly target K-Ras codon 12 mutants has been developed. It has been a great breakthrough in technology or research difficulty. However, tumors with Ras mutations are diverse. Thus, additional challenges will be faced in future research on Ras DNA.

## Author Contributions

F-YC and WH contributed to the conception and design of the study. F-YC organized the database, performed the statistical analysis, and wrote the first draft of the manuscript. XL and H-PZ contributed to the manuscript revision. All authors read and approved the submitted version.

## Conflict of Interest

The authors declare that the research was conducted in the absence of any commercial or financial relationships that could be construed as a potential conflict of interest.

## References

[B1] Abbott J. R. Hodges T. R. Daniels R. N. Patel P. A. Kennedy J. P. Howes J. E. (2018). Discovery of aminopiperidine indoles that activate the guanine nucleotide exchange factor SOS1 and modulate RAS signaling. *J. Med. Chem.* 61 6002–6017. 10.1021/acs.jmedchem.8b00360 29856609

[B2] Archambault J. Chambers R. S. Korbor M. S. Ho Y. Cartier M. Bolotin D. (1997). An essential component of a C-terminal domain phosphatase that interacts with transcription factor IIF in *Saccharomyces cerevisiae*. *Proc. Natl. Acad. Sci. U.S.A.* 94 14300–14305. 10.1073/pnas.94.26.14300 9405607 PMC24951

[B3] Bando T. Suqiyama S. (2006). Synthesis and biological properties of sequence-specific DNA-alkylating pyrrole-imidazole polyamides. *Acc. Chem. Res.* 39 935–944. 10.1021/ar030287f 17176032

[B4] Banerjee S. Kong D. Wang Z. Bao B. Hillman G. G. Sarkar F. H. (2011). Attenuation of multi-targeted proliferation-linked signaling by 3,3′-diindolylmethane (DIM): from bench to clinic. *Mutat. Res.* 728 47–66. 10.1016/j.mrrev.2011.06.001 21703360 PMC4120774

[B5] Baron R. A. Casey P. J. (2004). Analysis of the kinetic mechanism of recombinant human isoprenylcysteine carboxylmethyltransferase (Icmt). *BMC Biochem.* 5:19. 10.1186/1471-2091-5-19 15625008 PMC545952

[B6] Baron R. A. Peterson Y. K. Otto J. C. Rudolph J. Casey P. J. (2007). Time-dependent inhibition of isoprenylcysteine carboxyl methyltransferase by indole-based small molecules. *Biochemistry* 46 554–560. 10.1021/bi060344n 17209565

[B7] Baumli S. Lolli G. Lowe E. D. Troiani S. Rusconi L. Bullock A. N. (2008). The structure of P-TEFb (CDK9/cyclin T1), its complex with flavopiridol and regulation by phosphorylation. *EMBO J.* 27 1907–1918. 10.1038/emboj.2008.121 18566585 PMC2486423

[B8] Bergo M. O. Gavino B. J. Hong C. Beigneux A. P. McMahon M. Casey P. J. (2004). Inactivation of Icmt inhibits transformation by oncogenic K-Ras and B-Raf. *J. Clin. Invest.* 113 539–550. 10.1172/JCI18829 14966563 PMC338259

[B9] Bergo M. O. Leung G. K. Ambroziak P. Otto J. C. Casey P. J. Young S. G. (2000). Targeted inactivation of the isoprenylcysteine carboxyl methyltransferase gene causes mislocalization of K-Ras in mammalian cells. *J. Biol. Chem.* 275 17605–17610. 10.1074/jbc.C000079200 10747846

[B10] Bird G. Zorio D. A. Bentley D. L. (2004). RNA polymerase II carboxy-terminal domain phosphorylation is required for cotranscriptional pre-mRNA splicing and 3′-end formation. *Mol. Cell. Biol.* 24 8963–8969. 10.1128/MCB.24.20.8963-8969.2004 15456870 PMC517882

[B11] Burns M. C. Howes J. E. Sun Q. Little A. J. Camper D. V. Abbott J. R. (2018). High-throughput screening identifies small molecules that bind to the RAS:SOS:RAS complex and perturb RAS signaling. *Anal. Biochem.* 548 44–52. 10.1016/j.ab.2018.01.025 29444450 PMC5935105

[B12] Burns M. C. Sun Q. Daniels R. N. Camper D. Kennedy J. P. Phan J. (2014). Approach for targeting Ras with small molecules that activate SOS-mediated nucleotide exchange. *Proc. Natl. Acad. Sci. U.S.A.* 111 3401–3406. 10.1073/pnas.1315798111 24550516 PMC3948241

[B13] Chao S. H. Price D. H. (2001). Flavopiridol inactivates P-TEFb and blocks most RNA polymerase II transcription in vivo. *J. Biol. Chem.* 276 31793–31799. 10.1074/jbc.m102306200 11431468

[B14] Chen R. Keating M. J. Gandhi V. Plunkett W. (2005). Transcription inhibition by flavopiridol: mechanism of chronic lymphocytic leukemia cell death. *Blood* 106 2513–2519. 10.1182/blood-2005-04-1678 15972445 PMC1895272

[B15] Chripkova M. Zigo F. Mojzis J. (2016). Antiproliferative effect of indole phytoalexins. *Molecules* 21:1626. 10.3390/molecules21121626 27898039 PMC6274154

[B16] Clarke S. Tamanoi F. (2004). Fighting cancer by disrupting C-terminal methylation of signaling proteins. *J. Clin. Invest.* 113 513–515. 10.1172/JCI21059 14966560 PMC338270

[B17] Cox A. D. Fesik S. W. Kimmelman A. C. Luo J. Der C. J. (2014). Drugging the undruggable RAS: mission possible? *Nat. Rev. Drug Discov.* 13 828–851. 10.1038/nrd4389 25323927 PMC4355017

[B18] Dervan P. B. (2001). Molecular recognition of DNA by small molecules. *Bioorg. Med. Chem.* 9 2215–2235. 10.1016/s0968-0896(01)00262-0 11553460

[B19] Dolma S. Lessnick S. L. Hahn W. C. Stockwell B. R. (2003). Identification of genotype-selective antitumor agents using synthetic lethal chemical screening in engineered human tumor cells. *Cancer Cell* 3 285–296. 10.1016/s1535-6108(03)00050-3 12676586

[B20] Elhalem E. Donadio L. G. Zhou X. Lewin N. E. Garcia L. C. Lai C. C. (2017). Exploring the influence of indololactone structure on selectivity for binding to the C1 domains of PKCalpha, PKCepsilon, and RasGRP. *Bioorg. Med. Chem.* 25 2971–2980. 10.1016/j.bmc.2017.03.022 28392275 PMC5493039

[B21] Freedman T. S. Sondermann H. Firedland G. D. Kortemme T. Bar-Saqi D. Marqusee S. (2006). A Ras-induced conformational switch in the Ras activator Son of sevenless. *Proc. Natl. Acad. Sci. U.S.A.* 103 16692–16697. 10.1073/pnas.0608127103 17075039 PMC1629002

[B22] Garcia L. C. Donadio L. G. Mann E. Kolusheva S. Kedei N. Lewin N. E. (2014). Synthesis, biological, and biophysical studies of DAG-indololactones designed as selective activators of RasGRP. *Bioorg. Med. Chem.* 22 3123–3140. 10.1016/j.bmc.2014.04.024 24794745 PMC4104769

[B23] Go M. L. Leow J. L. Gorla S. K. Schuller A. P. Wang M. Casey P. J. (2010). Amino derivatives of indole as potent inhibitors of isoprenylcysteine carboxyl methyltransferase. *J. Med. Chem.* 53 6838–6850. 10.1021/jm1002843 20809634

[B24] Gottesfeld J. M. Turner J. M. Dervan P. B. (2000). Chemical approaches to control gene expression. *Gene Expr.* 9 7–91. 10.3727/000000001783992696 PMC5964961 11097426

[B25] Guo W. Wu S. Liu J. Fang B. (2008). Identification of a small molecule with synthetic lethality for K-ras and protein kinase C iota. *Cancer Res.* 68 7403–7408. 10.1158/0008-5472.can-08-1449 18794128 PMC2678915

[B26] Guo W. Wu S. Wang L. Wang R. Y. Wei X. Liu J. (2009). Interruption of RNA processing machinery by a small compound, 1-[(4-chlorophenyl)methyl]-1H-indole-3-carboxaldehyde (oncrasin-1). *Mol. Cancer Ther.* 8 441–448. 10.1158/1535-7163.mct-08-0839 19208825 PMC2653085

[B27] Guo W. Wu S. Wang L. Wei X. Liu X. Wang J. (2011). Antitumor activity of a novel oncrasin analogue is mediated by JNK activation and STAT3 inhibition. *PLoS ONE* 6:e28487. 10.1371/journal.pone.0028487 22174819 PMC3236185

[B28] Haque A. Rahman M. A. Faizi M. S. H. Khan M. S. (2018). Next generation antineoplastic agents: a review on structurally modified vinblastine (VBL) analogues. *Curr. Med. Chem.* 25 1650–1662. 10.2174/0929867324666170502123639 28464783

[B29] Hashmi M. A. Khan A. Farooq U. Khan S. (2018). Alkaloids as cyclooxygenase inhibitors in anticancer drug discovery. *Curr. Protein Pept. Sci.* 19 292–301. 10.2174/1389203718666170106103031 28059042

[B30] Hiraoka K. Inoue T. Taylor R. D. Watanabe T. Koshikawa N. Yoda H. (2015). Inhibition of KRAS codon 12 mutants using a novel DNA-alkylating pyrrole-imidazole polyamide conjugate. *Nat. Commun.* 6:6706. 10.1038/ncomms7706 25913614

[B31] Howes J. E. Akan D. T. Burns M. C. Rossanese O. W. Waterson A. G. Fesik S. W. (2018). Small molecule-mediated activation of RAS elicits biphasic modulation of phospho-ERK levels that are regulated through negative feedback on SOS1. *Mol. Cancer. Ther.* 17 1051–1060. 10.1158/1535-7163.mct-17-0666 29440291

[B32] Huang Y. Tan H. Guo Z. Wu X. Zhang Q. Zhang L. (2016). The biosynthesis and genetic engineering of bioactive indole alkaloids in plants. *J. Plant Biol.* 59 203–214. 10.1007/s12374-016-0032-5

[B33] Hunter J. C. Manandhar A. Carrasco M. A. Gurbani D. Gondi S. Westover K. D. (2015). Biochemical and structural analysis of common cancer-associated KRAS mutations. *Mol. Cancer Res.* 13 1325–1335. 10.1158/1541-7786.mcr-15-0203 26037647

[B34] Hurdle J. G. O’Neill A. J. Chopra I. (2004). Anti-staphylococcal activity of indolmycin, a potential topical agent for control of staphylococcal infections. *J. Antimicrob Chemother* 54 549–552. 10.1093/jac/dkh352 15243028

[B35] Järvisalo J. Saris N. E. (1975). Action of propranolol on mitochondrial functions–effects on energized ion fluxes in the presence of valinomycin. *Biochem. Pharmacol.* 24 1701–1705. 10.1016/0006-2952(75)90009-x 13

[B36] Karaquni I. M. Glüsenkamp K. H. Langerak A. Geisen C. Ullrich V. Winde G. (2002a). New indene-derivatives with anti-proliferative properties. *Bioorg. Med. Chem. Lett.* 12 709–713. 10.1016/s0960-894x(01)00839-3 11844707

[B37] Karaquni I. M. Herter P. Debruyne P. Chtarbova S. Kasprzynski A. Herbrand U. (2002b). The new sulindac derivative IND 12 reverses Ras-induced cell transformation. *Cancer Res.* 62 1718–1723. 11912145

[B38] Kim E. Du L. Breqman B. Warren S. L. (1997). Splicing factors associate with hyperphosphorylated RNA polymerase II in the absence of pre-mRNA. *J. Cell. Biol.* 136 19–28. 10.1083/jcb.136.1.19 9008700 PMC2132468

[B39] Lau H. Y. Ramanujulu P. M. Guo D. Yang T. Wirawan M. Casey P. J. (2014). An improved isoprenylcysteine carboxylmethyltransferase inhibitor induces cancer cell death and attenuates tumor growth in vivo. *Cancer Biol. Ther.* 15 1280–1291. 10.4161/cbt.29692 24971579 PMC4128870

[B40] Lim S. M. Westover K. D. Ficarro S. B. Harrison R. A. Choi H. G. Pacold M. E. (2014). Therapeutic targeting of oncogenic K-Ras by a covalent catalytic site inhibitor. *Angew. Chem. Int. Ed. Engl.* 53 199–204. 10.1002/anie.201307387 24259466 PMC3914205

[B41] Liu J. Yang G. Thompson-Lanza J. A. Glassman A. Hayes K. Patterson A. (2004). A genetically defined model for human ovarian cancer. *Cancer Res.* 64 1655–1663. 10.1158/0008-5472.can-03-3380 14996724

[B42] Luo J. Emanuele M. J. Li D. Creighton C. J. Schlabach M. R. Westbrook T. F. (2009). A genome-wide RNAi screen identifies multiple synthetic lethal interactions with the Ras oncogene. *Cell* 137 835–848. 10.1016/j.cell.2009.05.006 19490893 PMC2768667

[B43] MacCallum D. E. Melville J. Frame S. Watt K. Anderson S. Gianella-Borradori A. (2005). Seliciclib (CYC202, R-Roscovitine) induces cell death in multiple myeloma cells by inhibition of RNA polymerase II-dependent transcription and down-regulation of Mcl-1. *Cancer Res.* 65 5399–5407. 10.1158/0008-5472.can-05-0233 15958589

[B44] Maurer T. Garrenton L. S. Oh A. Pitts K. Anderson D. J. Skelton N. J. (2012). Small-molecule ligands bind to a distinct pocket in Ras and inhibit SOS-mediated nucleotide exchange activity. *Proc. Natl. Acad. Sci. U.S.A.* 109 5299–5304. 10.1073/pnas.1116510109 22431598 PMC3325706

[B45] McCracken S. Fong N. Yankulov K. Ballantyne S. Pan G. Greenblatt J. (1997). The C-terminal domain of RNA polymerase II couples mRNA processing to transcription. *Nature* 385 357–361. 10.1038/385357a0 9002523

[B46] Millhouse S. Manley J. L. (2005). The C-terminal domain of RNA polymerase II functions as a phosphorylation-dependent splicing activator in a heterologous protein. *Mol. Cell Biol.* 25 533–544. 10.1128/MCB.25.2.533-544.2005 15632056 PMC543425

[B47] Misteli T. Spector D. L. (1999). RNA polymerase II targets pre-mRNA splicing factors to transcription sites in vivo. *Mol. Cell* 3 697–705. 10.1016/s1097-2765(01)80002-2 10394358

[B48] Montserrat-de la Paz S. Fernandez-Arche A. de la Puerta R. Quilez A. M. Muriana F. J. Garcia-Gimenez M. D. (2016). Mitraphylline inhibits lipopolysaccharide-mediated activation of primary human neutrophils. *Phytomedicine* 23 141–148. 10.1016/j.phymed.2015.12.015 26926175

[B49] Nalamachu S. Wortmann R. (2014). Role of indomethacin in acute pain and inflammation management: a review of the literature. *Postgrad. Med.* 126 92–97. 10.3810/pgm.2014.07.2787 25141247

[B50] Okada M. Sugita T. Wong C. P. Wakimoto T. Abe I. (2017). Identification of pyridinium with three indole moieties as an antimicrobial agent. *J. Nat. Prod.* 80 1205–1209. 10.1021/acs.jnatprod.6b01152 28290701

[B51] Ostrem J. M. Peters U. Sos M. L. Wells J. A. Shokat K. M. (2013). K-Ras(G12C) inhibitors allosterically control GTP affinity and effector interactions. *Nature* 503 548–551. 10.1038/nature12796 24256730 PMC4274051

[B52] Patil R. Patil S. A. Beaman K. D. Patil S. A. (2016). Indole molecules as inhibitors of tubulin polymerization: potential new anticancer agents, an update (2013-2015). *Future Med. Chem.* 8 1291–1316. 10.4155/fmc-2016-0047 27476704

[B53] Patricelli M. P. Janes M. R. Li L. S. Hansen R. Peters U. Kessler L. V. (2016). Selective inhibition of oncogenic KRAS output with small molecules targeting the inactive state. *Cancer Discov.* 6 316–329. 10.1158/2159-8290.cd-15-1105 26739882

[B54] Ramanujulu P. M. Yang T. Yap S. Q. Wong F. C. Casey P. J. Wang M. (2013). Functionalized indoleamines as potent, drug-like inhibitors of isoprenylcysteine carboxyl methyltransferase (Icmt). *Eur. J. Med. Chem.* 63 378–386. 10.1016/j.ejmech.2013.02.007 23514631

[B55] Reynolds V. L. McGovern J. P. Hurley L. H. (1986). The chemistry, mechanism of action and biological properties of CC-1065, a potent antitumor antibiotic. *J. Antibiot. (Tokyo)* 39 319–334. 10.7164/antibiotics.39.319 3516958

[B56] Scholl C. Frohling S. Dunn I. F. Schinzel A. C. Barbie D. A. Kim S. Y. (2009). Synthetic lethal interaction between oncogenic KRAS dependency and STK33 suppression in human cancer cells. *Cell* 137 821–834. 10.1016/j.cell.2009.03.017 19490892

[B57] Sharma A. Fonseca L. L. Rajani C. Yanagida J. K. Endo Y. Cline J. M. (2014). Targeted deletion of RasGRP1 impairs skin tumorigenesis. *Carcinogenesis* 35 1084–1091. 10.1093/carcin/bgu016 24464785 PMC4004207

[B58] Shi Y. Q. Rando R. R. (1992). Kinetic mechanism of isoprenylated protein methyltransferase. *J. Biol. Chem.* 267 9547–9551. 1577795

[B59] Shima F. Yoshikawa Y. Ye M. Araki M. Matsumoto S. Liao J. (2013). In silico discovery of small-molecule Ras inhibitors that display antitumor activity by blocking the Ras-effector interaction. *Proc. Natl. Acad. Sci. U.S.A.* 110 8182–8187. 10.1073/pnas.1217730110 23630290 PMC3657810

[B60] Singh A. K. Raj V. Saha S. (2017). Indole-fused azepines and analogues as anticancer lead molecules: privileged findings and future directions. *Eur. J. Med. Chem.* 142 244–265. 10.1016/j.ejmech.2017.07.042 28803677

[B61] Song X. Lopez-Campistrous A. Sun L. Dower N. A. Kedei N. Yang J. (2013). RasGRPs are targets of the anti-cancer agent ingenol-3-angelate. *PLoS ONE* 8:e72331. 10.1371/journal.pone.0072331 23991094 PMC3749120

[B62] Stone J. C. (2011). Regulation and function of the RasGRP family of ras activators in blood cells. *Genes Cancer* 2 320–334. 10.1177/1947601911408082 21779502 PMC3128638

[B63] Sun Q. Burke J. P. Phan J. Burns M. C. Olejniczak E. T. Waterson A. G. (2012). Discovery of small molecules that bind to K-Ras and inhibit Sos-mediated activation. *Angew. Chem. Int. Ed. Engl.* 51 6140–6143. 10.1002/anie.201201358 22566140 PMC3620661

[B64] Tamanoi F. Lu J. (2013). Recent progress in developing small molecule inhibitors designed to interfere with ras membrane association: toward inhibiting K-Ras and N-Ras functions. *Enzymes* 34(Pt. B), 181–200. 10.1016/b978-0-12-420146-0.00008-1 25034105

[B65] Taveras A. G. Remiszewski S. W. Doll R. J. Cesarza D. Huang E. C. Kirschmeier P. (1997). Ras oncoprotein inhibitors: the discovery of potent, ras nucleotide exchange inhibitors and the structural determination of a drug-protein complex. *Bioorg. Med. Chem.* 5 125–133. 10.1016/s0968-0896(96)00202-7 9043664

[B66] Taylor R. D. Asamitsu S. Takenaka T. Yamamoto M. Hashiya K. Kawamoto Y. (2014). Sequence-specific DNA alkylation targeting for Kras codon 13 mutation by pyrrole-imidazole polyamide seco-CBI conjugates. *Chemistry* 20 1310–1317. 10.1002/chem.201303295 24382626

[B67] Torrance C. J. Agrawal V. Voqelstein B. Kinzier K. W. (2001). Use of isogenic human cancer cells for high-throughput screening and drug discovery. *Nat. Biotechnol.* 19 940–945. 10.1038/nbt1001-940 11581659

[B68] Wang Y. D. Dziegielewski J. Chang A. Y. Dervan P. B. Beerman T. A. (2002). Cell-free and cellular activities of a DNA sequence selective hairpin polyamide-CBI conjugate. *J. Biol. Chem.* 277 42431–42437. 10.1074/jbc.M207179200 12196541

[B69] Watanabe H. Rose M. T. Aso H. (2011). Role of peripheral serotonin in glucose and lipid metabolism. *Curr. Opin. Lipidol.* 22 186–191. 10.1097/MOL.0b013e3283462273 21494145

[B70] White A. W. Carpenter N. Lottin J. R. McClelland R. A. Nicholson R. I. (2012). Synthesis and evaluation of novel anti-proliferative pyrroloazepinone and indoloazepinone oximes derived from the marine natural product hymenialdisine. *Eur. J. Med. Chem.* 56 246–253. 10.1016/j.ejmech.2012.08.022 22995819

[B71] Whitehurst A. W. Bodemann B. O. Cardenas J. Ferguson D. Girard L. Peyton M. (2007). Synthetic lethal screen identification of chemosensitizer loci in cancer cells. *Nature* 446 815–819. 10.1038/nature05697 17429401

[B72] Winter-Vann A. M. Baron R. A. Wong W. dela Cruz J. York J. D. Gooden D. M. (2005). A small-molecule inhibitor of isoprenylcysteine carboxyl methyltransferase with antitumor activity in cancer cells. *Proc. Natl. Acad. Sci. U.S.A.* 102 4336–4341. 10.1073/pnas.0408107102 15784746 PMC555472

[B73] Wu S. Wang L. Guo W. Liu X. Liu J. Wei X. (2011). Analogues and derivatives of oncrasin-1, a novel inhibitor of the C-terminal domain of RNA polymerase II and their antitumor activities. *J. Med. Chem.* 54 2668–2679. 10.1021/jm101417n 21443218 PMC3082393

[B74] Yang M. C. Peng C. Huang H. Yang L. He X. H. Huang W. (2017). Organocatalytic asymmetric synthesis of spiro-oxindole piperidine derivatives that reduce cancer cell proliferation by inhibiting MDM2-p53 interaction. *Org. Lett.* 19 6752–6755. 10.1021/acs.orglett.7b03516 29210587

[B75] Yu C. P. Song Y. L. Zhu Z. M. Huang B. Xiao Y. Q. Luo D. Y. (2017). Targeting TDO in cancer immunotherapy. *Med. Oncol.* 34:73. 10.1007/s12032-017-0933-2 28357780

[B76] Yu H. Zhang H. Dong M. Wu Z. Shen Z. Xie Y. (2017). Metabolic reprogramming and AMPKalpha1 pathway activation by caulerpin in colorectal cancer cells. *Int. J. Oncol.* 50 161–172. 10.3892/ijo.2016.3794 27922662

[B77] Zhao Q. Peng C. Huang H. Liu S. J. Zhong Y. J. Huang W. (2018). Asymmetric synthesis of tetrahydroisoquinoline-fused spirooxindoles as Ras-GTP inhibitors that inhibit colon adenocarcinoma cell proliferation and invasion. *Chem. Commun. (Camb)* 54 8359–8362. 10.1039/c8cc04732d 29993051

